# Examining Brain Structural Connectivity in Early-life Interpersonal Stress

**DOI:** 10.1192/j.eurpsy.2023.478

**Published:** 2023-07-19

**Authors:** L. Lim, L. Talozzi, H. Howells

**Affiliations:** ^1^ Singapore Institute for Clinical Sciences (SICS), Agency for Science, Technology and Research (ASTAR); ^2^Neuroscience & Mental Health, Lee Kong Chian School of Medicine, Nanyang Technological University Singapore, Singapore; ^3^Child & Adolescent Psychiatry, IOPPN, King’s College London, London, United Kingdom; ^4^Neurology and Neurological Sciences, Stanford University, California, United States; ^5^Department of Medical Biotechnology and Translational Medicine, University of Milan and Humanitas Research Hospital, Milan, Italy

## Abstract

**Introduction:**

Early-life interpersonal stress, particularly childhood maltreatment (CM), is associated with social cognition deficits as well as neurobiological abnormalities including alterations in brain structure and function and heightened inflammation. However, few studies have investigated whether peer victimisation (PV) has similar effects.

**Objectives:**

This study first examines the associations between white matter tract abnormalities and childhood interpersonal stress from carers (CM) and peers (PV). Next, it explores how the observed tract alterations are in terms related to cytokine IL-6 level and theory of mind (ToM) performance in the CM and PV groups.

**Methods:**

Data were collected from 107 age-and gender-matched youths (34 CM, 35 PV and 38 controls). Tractography and whole-brain tract-based spatial statistics (TBSS) analyses were conducted.

**Results:**

Tractography showed that both CM and PV groups had smaller right inferior longitudinal fasciculus (ILF) tract volume than controls, which was furthermore associated with longer maltreatment duration within the CM group. At the microstructural level, the CM group had higher fractional anisotropy (FA) of bilateral anterior thalamic radiation (ATR) than both PV and control groups, which were associated with enhanced affective ToM performance in maltreated individuals only. Reduced left ATR FA, however, was associated with greater emotional and behavioural difficulties in the PV group. Using TBSS, the CM group had higher FA than both PV and control groups in predominantly right-hemispheric limbic tracts (UF, ATR, ILF, cingulum bundle and inferior fronto-occipital fasciculus), corpus callosum and corona radiata, which were furthermore associated with heightened cytokine IL-6 level within the CM group.

**Conclusions:**

Early-life interpersonal stress, particularly from carers, is associated with widespread alterations of neural pathways connecting the frontal, temporal and occipital cortices involved in cognitive and affective control. The adverse caregiving experience may conceivably contribute to enhanced mental-state decoding but there exists a hidden cost of heightened inflammation in these seemingly healthy maltreated youths. Our findings thus underscore the need to further examine the mental and physical well-being of healthy individuals exposed to early-life stress as they may still be vulnerable to psychopathology later on.

**Disclosure of Interest:**

None Declared

